# Preparation of Cassia Bean Gum/Soy Protein Isolate Composite Matrix Emulsion Gel and Its Effect on the Stability of Meat Sausage

**DOI:** 10.3390/gels10100643

**Published:** 2024-10-09

**Authors:** Qiang Zou, Yuhan Zheng, Yudie Liu, Linghui Luo, Yuyou Chen, Guilian Ran, Dayu Liu

**Affiliations:** 1 School of Food and Biological Engineering, Chengdu University, Chengdu 610106, China; zouqiang@cdu.edu.cn (Q.Z.); zhengyuhan@stu.cdu.edu.cn (Y.Z.); liuyudie@stu.cdu.edu.cn (Y.L.); luolinghui@stu.cdu.edu.cn (L.L.); chenyuyou@stu.cdu.edu.cn (Y.C.); ranguilian@stu.cdu.edu.cn (G.R.); 2 Meat Processing Key Laboratory of Sichuan Province, School of Food and Biological Engineering, Chengdu University, Chengdu 610106, China

**Keywords:** composite matrix emulsion gel, cassia bean gum, meat and sausage, fat substitute, freeze–thaw stability

## Abstract

The use of plant-derived emulsified gel systems as fat substitutes for meat products has always been an important direction in the development of healthy foods. In this study, a composite matrix emulsion gel was prepared with soy protein isolate (SPI) and different concentrations of cassia bean gum (CG), and then the selected emulsion gel was applied to meat sausage as a fat substitute to explore its stability. Our results showed that the hardness, chewiness, viscosity, shear stress, and G′ and G″ moduli of the emulsion gel increased considerably with the cassia bean gum concentration, the thickness of the emulsion gel increased, and the pore size decreased. The gel strength of the 1.75% CG/SPI emulsion gel was the highest, which was 586.91 g. The elasticity was 0.94 mm, the masticability was 452.94 mJ, and the water-holding capacity (WHC) was 98.45%. Then, the 1.75% CG/SPI emulsion gel obtained via screening was applied as a fat substitute in meat sausage. With an increase in the substitution amount, the cooking loss, emulsification stability, pH, color difference, texture, and antioxidant activity of the meat sausage before and after freezing and thawing increased first and then decreased. The indexes of meat sausage with 50% fat replacement were not considerably different from those of full-fat meat sausage. This study can provide a theoretical basis for the application of plant-derived emulsified gel systems as fat substitutes in meat sausage.

## 1. Introduction

Meat sausage is a popular and frequently eaten processed meat product because of its high nutritional value, convenience, and unique flavor. Pig back fat is one of the main raw materials of meat sausage (accounting for approximately 30%). It not only improves the flavor of meat sausage but also affects its texture and sensory quality. However, excessive intake will increase the risk of diseases, such as coronary heart disease, cardiovascular disease, diabetes, and obesity [1], adversely affecting human health. In addition, traditional meat sausage retains oil and has poor freeze–thaw and oxidation stability because of excessive animal fat [2]. Thus, the meat industry has studied different lipid reduction technology strategies to produce healthier and more nutritious products while improving product stability.

Emulsion gels as animal fat substitutes have gradually attracted interest. They are gel-like solid materials with spatial network structures that are formed through an emulsion that is initiated using induction methods, which can effectively emulsify and disperse oil droplets [3]. The preparation of emulsion gels with proteins or polysaccharides often has limited practical applications because of the poor emulsification and thermal stability of the products [4]. Hence, many studies have focused on preparing composite matrix emulsion gels with excellent properties, not only enhancing the emulsifying effect of the emulsions but also improving the stability of the emulsion gels [5].

Soy protein isolate is one of the high-quality plant proteins that can replace animal protein, mainly consisting of soy globulin and β-conglycinin, and can be applied to foods to increase their protein content and provide desired functions such as gelation, emulsification, and foaming properties, where the ability to gel is a key functional property of SPI. The gelation process begins with protein denaturation, resulting in structural changes, the exposure of functional groups including sulfhydryl or hydrophobic groups, and then various interactions that trigger aggregation [6]. Moreover, the incorporation of polysaccharides can alter the intermolecular and/or intramolecular interactions involved in the complex, thus affecting the gelling function of the protein [7]. Zhang et al. [8] prepared emulsion gels containing soy protein isolate and pectin using the thermal sensitivity method, which effectively improved the texture, rheology, and microscopic and other functional properties of composite gels. For novel food development, protein–polysaccharide interactions are widely used to achieve certain food functions. Wang et al. [9] added an emulsion gel, which was prepared via the composite preparation of soy protein isolate and κ-carrageenan, to sausage in different proportions instead of pig backfat to reduce the content of fat and cholesterol, and the difference in texture and product yield was not significant compared with the control group. In this regard, there are few reports on emulsion gels being prepared using the combination of soybean protein isolate and cassia bean gum. Cassia bean gum is the polysaccharide of cassia seeds. It is a water-soluble polysaccharide that is extracted and isolated from the endosperm of mature seeds of Cassia obtusifolia or small cassia in the legumes. Its main structure is composed of galactomannan. It has been reported that cassia bean gum can be used as a fat substitute in food and can achieve thickening, freeze–thaw stability, synergistic gelatinization, film formation, etc. [10]. Cao et al. [11] made an edible oil packaging film from cassia bean gum, which has a higher barrier property and heat-sealing property. There are few reports on the application of cassia bean gum as a fat substitute. Generally, meat sausage is a frozen storage food, and its freeze–thaw stability and storage stability have an important impact on the product quality. However, the application of soy protein isolate/cassia bean gum composite matrix emulsion gel as a fat substitute in meat sausage has not been studied.

Therefore, in this study, soybean protein isolate and cassia bean gum were prepared into a protein–polysaccharide composite matrix emulsion gel using heat treatment, and the effects of cassia bean gum (0%, 0.85%, 1.15%, 1.45%, 1.75%, and 2%) at different concentrations on the rheological behavior, texture properties, microstructure, WHC, and thermal phase transformation temperature of the composite matrix emulsion gel were investigated. Then, the selected emulsion gel was used as a fat substitute and added into meat sausage at different replacement amounts (0%, 25%, 50%, 75%, and 100%). The texture, emulsification stability, color difference, and antioxidant activity during the storage of meat sausage before and after freezing and thawing were used as indicators to screen out the meat sausage with the best stable fat replacement amount. This study serves as a reference for the research and development of low-fat meat sausage products. 

## 2. Results and Discussion

### 2.1. Texture Characteristics of the Emulsion Gel

As shown in Table 1, the hardness and chewiness of the emulsion gel increased with the CG concentration, increasing from 43.67 N to 586.91 N and 43.53 mJ to 452.94 mJ, respectively. When the CG concentration was greater than 1.75%, the properties decreased to 558.94 N and 339.89 mJ, respectively. Cassia bean gum may have promoted the interaction between the proteins and polysaccharides, forming a dense gel network structure [12]. Cassia bean gum at 2% reduced firmness and chewiness because the overfilling effect inhibited the aggregation of SPIs. This result is consistent with the previous results of Jiang et al.’s study [13] on the effect of κ-carrageenan on the texture of oyster protein treated with high pressure homogenization.

The initial elasticity and cohesion of the emulsion gel were 0.99 and 0.98, respectively (Table 1). As the CG concentration increased, the values gradually declined. The lowest values were 0.93 and 0.80, respectively, and the gradual decrease in cohesiveness indicated that damage to the irreversible structure of the sample increased after compression. The elasticity decreased, but the change was not considerable. The high chewiness of the sample indicated that the emulsion gel was mainly elastic. This may be because the electrostatic attraction between soy protein isolate increases the interface adsorption, protein aggregation enhances the viscoelasticity of the gel, and the addition of an appropriate polysaccharide increases the viscosity of the continuous phase; the structure of the emulsion gel is more stable, and the polysaccharide does not affect the protein-led gel behavior in the emulsion gel [14].

### 2.2. Rheological Behavior of the Emulsion Gel

The shear rate ranged from 0.01 s^−1^ to 10 s^−1^, the viscosity of the emulsion gel decreased with an increasing shear rate, and the emulsion gel showed typical pseudoplastic fluid characteristics [15]. Different concentrations of CG showed similar effects on the emulsion gel’s viscosity, and shear thinning behavior was observed at all concentrations possibly because the gel network structure in the emulsion was destroyed by shear force. Moreover, droplet migration resistance was reduced, and this effect prevented the dispersion and aggregation of oil droplets and decreased the apparent viscosity [16]. At the same shear rate, compared with the 0% CG/SPI emulsion gel, the emulsion gel with a higher CG concentration had higher viscosity, and the 2% CG/SPI emulsion gel had the highest viscosity possibly due to the cross-linking of CG and SPI after heating. This process increased the viscosity of the gel network structure and, thus, reduced the degree of shear thinning [17].

**Figure 1:**
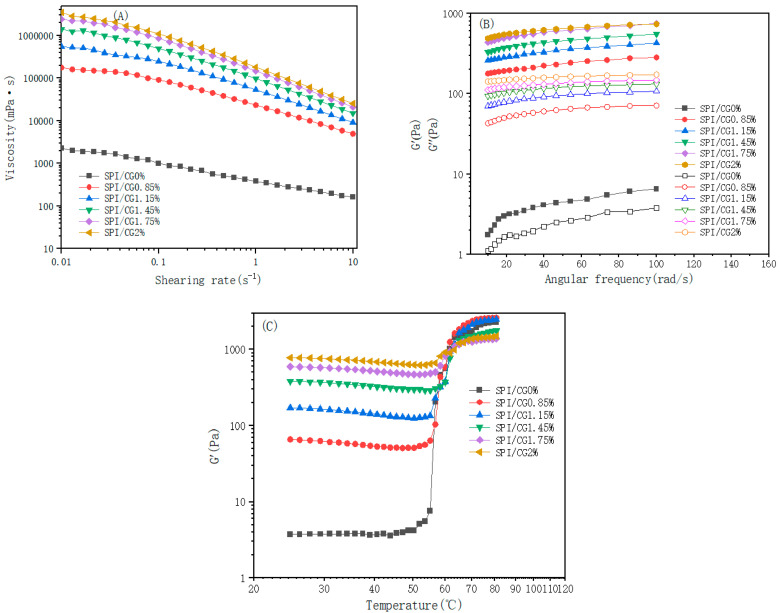
Rheological characteristics of emulsion gels with different concentrations of cassia bean gum. Effects of cassia bean gum on the viscosity (**A**), frequency scan (**B**), and temperature scan (**C**). Data are expressed as the mean ± standard deviation. SPI/CG 0%: soy protein isolate, no cassia bean gum added; SPI/CG 0.85%: SPI + 0.85% cassia bean gum; SPI/CG 1.15%: SPI + 1.15% cassia bean gum; SPI/CG 1.45%: SPI + 1.45% cassia bean gum; SPI/CG 1.75%: SPI + 1.75% cassia bean gum; SPI/CG 2%: SPI + 2% cassia bean gum. Different letters indicate significant differences in the data (*p* < 0.05).

The energy storage modulus (G′) and consumption modulus (G″) of emulsion gels with different concentrations of CG varied within a frequency range of 10–100 rad/s (Figure 1B). Different concentrations of cassia bean gum increased the G′ values of the emulsion gels to different degrees, and the G′ value increased with the cassia bean gum concentration. These changes indicated that the G′ value of an emulsion gel was less dependent on the application frequency, and the network structure of the gel was stable and not easily destroyed [18]. In addition, the G′ values of the emulsion gels with different concentrations of CG were higher than the G″ values, indicating that the emulsion gels were mainly elastic gel network structures. The 2% CG/SPI emulsion gels had large G′ and G″ values and prominent viscoelastic properties possibly due to the noncovalent interactions between cassia bean gum and proteins as anion hydrophilic colloids. The flow of free water in the gel network was restricted, and the gel network was enhanced [19].

Figure 1C shows the change in the G′ value of the SPI/CG emulsion gel at varying temperatures. The change in the G′ values of the composite gels presented two stages during heating. At 25–53 °C, the G′ value tended to be stable at an increasing temperature. The G′ value of the emulsion gels at this stage was much higher than that of the 0% CG/SPI emulsion gels, and the G′ value increased with the CG concentration. The interaction of hydrogen or ionic bonds between CG and SPI in the gel network enhanced the elasticity and hardness of the emulsion gel [20]. When the temperature was higher than 56 °C, the G′ value of the emulsion gel with different concentrations of CG increased considerably, and different degrees of “kinking” occurred on the G′ curve, that is, the melting and gelation behaviors of the gel, indicating that CG exerted a synergistic effect that enhanced the gel strength of the emulsion and helped to improve the final gel strength [21].

### 2.3. Water-Holding Capacity and Strength of the Emulsion Gel

**Figure 2:**
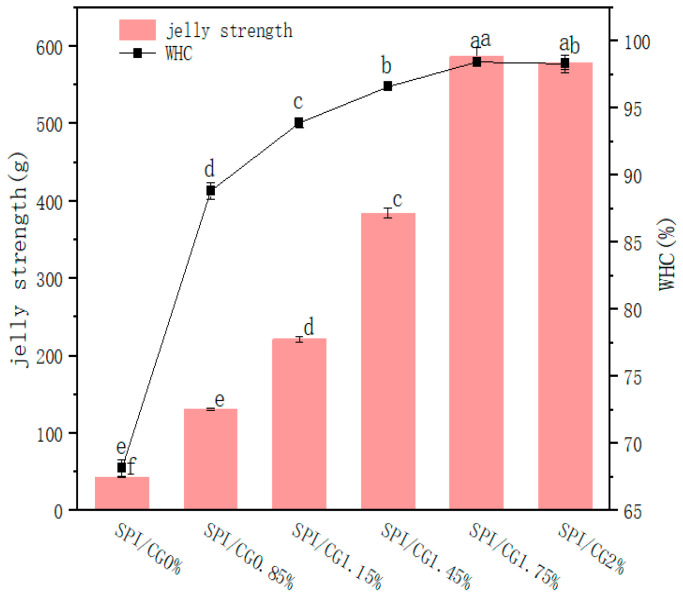
Effects of different concentrations of cassia bean gum on the water holding capacity and strength of emulsion gel. SPI/CG 0%: soy protein isolate, no cassia bean gum added; SPI/CG 0.85%: SPI + 0.85% cassia bean gum; SPI/CG 1.15%: SPI + 1.15% cassia bean gum; SPI/CG 1.45%: SPI + 1.45% cassia bean gum; SPI/CG 1.75%: SPI + 1.75% cassia bean gum; SPI/CG 2%: SPI + 2% cassia bean gum. Different letters indicate significant differences in the data (*p* < 0.05).

The gel strength can directly reflect the gelling degree of a complex gel, is a key index for evaluating the gel structures of proteins and protein-based products, and is closely related to the WHC. As shown in Figure 2, the gel strength and WHC increased first and then decreased with an increasing CG concentration. The gel strength increased from 43.67 g to 586.91 g, and the highest gel strength of the emulsion gel (586.91 g) was obtained at a 1.75% CG concentration. The presence of polysaccharides could affect the microstructure and texture properties of the gel, which might be related to an exclusion effect of incompatible biopolymers in a mixed solution [22]. Moreover, an increase in the CG concentrations led to the increase in hydrogen-bonding groups, thus enhancing the internal network structure of the emulsion gels.

The WHC is one of the most important indexes for evaluating the structural quality of protein gel networks. The WHC increased from 68.17% to 98.45%, and a 30% increase in water retention rate was observed. These results are similar to those in Table 1. On the one hand, more and more CG filled the void space of the SPI, thus reducing the pore size of the gel network; on the other hand, the increase in CG concentrations led to the tighter adsorption of SPI with CG onto the oil–water interface, thus improving the compactness of the gel structure [23].

### 2.4. Observation of the Emulsion Gel Structure

**Figure 3:**
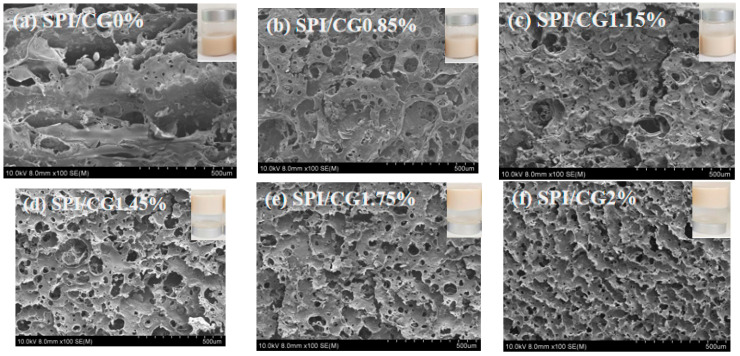
The appearance and scanning electron microscopy (SEM) images of emulsion gels with different concentrations of cassia bean gum. (**a**) SPI/CG 0%: soy protein isolate, no cassia bean gum added, (**b**) SPI/CG 0.85%: SPI + 0.85% cassia bean gum, (**c**) SPI/CG 1.15%: SPI + 1.15% cassia bean gum, (**d**) SPI/CG 1.45%: SPI + 1.45% cassia bean gum, (**e**) SPI/CG 1.75%: SPI + 1.75% cassia bean gum, and (**f**) SPI/CG 2%: SPI + 2% cassia bean gum.

As shown in Figure 3, the emulsion gels with different concentrations of CG were white, and the addition of 1.45% cassia bean gum resulted in the formation of a line dividing the different morphology of the composite gel. The addition of 1.45% or more CG can form a complete semisolid composite gel in positive and negative positions, whereas the composite emulsion gels with less than 1.45% CG were semifluid and semisolid. These results demonstrated that the cassia bean gum concentration plays an important role in emulsion gel formation, and smooth and delicate gels with hard surfaces can be obtained by increasing the cassia bean gum concentration.

The microstructure of the gels was observed using scanning electron microscopy. The 0% CG/SPI emulsion gel presented a relatively smooth and irregular network composed of cavities of different sizes. After cassia bean gum was added, the emulsion gel presented a network structure composed of pores of different sizes and flaky edges. The pores were a result of the synergistic effect of SPI and cassia bean gum, which formed a dense gel with a lamellar pore structure [24]. As the CG concentration increased, the size of the structural pores in the emulsion gel gradually decreased, whereas the thickness of the flake increased. Thus, the stability of the emulsion increased. This result was consistent with the observed appearance of the gel. The highest thickness of the emulsion gel and the smallest pores were obtained by adding 2% CG. This may be because during the gelation process of soy protein isolate, protein structures aggregate to form a three-dimensional gel network, and some denatalized, exposed functional groups interact with cassia gum to form a dense, saturated spatial network structure [25].

### 2.5. FTIR Spectroscopy of Emulsion Gel

**Figure 4:**
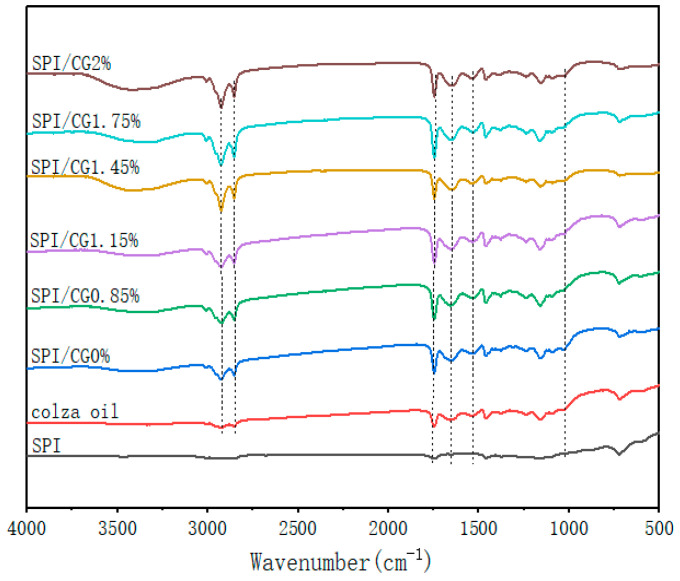
FTIR spectra of emulsion gels with different concentrations of cassia bean gum. SPI: pure soy protein isolate solution; SPI/CG 0%: soy protein isolate, no added cassia bean gum; SPI/CG 0.85%: SPI + 0.85% cassia bean gum; SPI/CG 1.15%: SPI + 1.15% cassia bean gum; SPI/CG 1.45%: SPI + 1.45% cassia bean gum; SPI/CG 1.75%: SPI + 1.75% cassia bean gum; SPI/CG 2%: SPI + 2% cassia bean gum.

The chemical interaction information of emulsion gels with different concentrations of cassia bean gum were investigated using FTIR spectroscopy (Figure 4). The wide absorption peak between 3100 and 3500 cm^−1^ was a response to hydrogen bonds (O-H and N-H; Figure 4). As the CG concentration increased, the peak value of the hydrogen bond gradually increased, and the strength of the hydrogen bonds was enhanced during gel formation [26]. These observations were consistent with previous reports on cassia bean gum and κC/KGM composite gels [27].

The strong absorption bands at 2928, 2854, 1748, and 1026 cm^−1^ were the characteristic signals of rapeseed oil, indicating that rapeseed oil only served as a filler in the emulsion gel, but the particle size of the oil droplets changed. The amide I band (1635.69 cm^−1^) was mainly caused by the C=O stretching vibration in the peptide bond, and the amide II band (1532.03 cm^−1^) represented the bending vibration of the N-H group and the stretching vibration of the C-N group. All SPI/CG emulsion gels showed an increased absorption peak strength at amides I and II possibly due to the large number of hydrogen bonds and cross-linked isopeptide bonds that were generated during gel formation [28].

### 2.6. Analysis of the Thermal Properties of the Emulsion Gel

**Figure 5:**
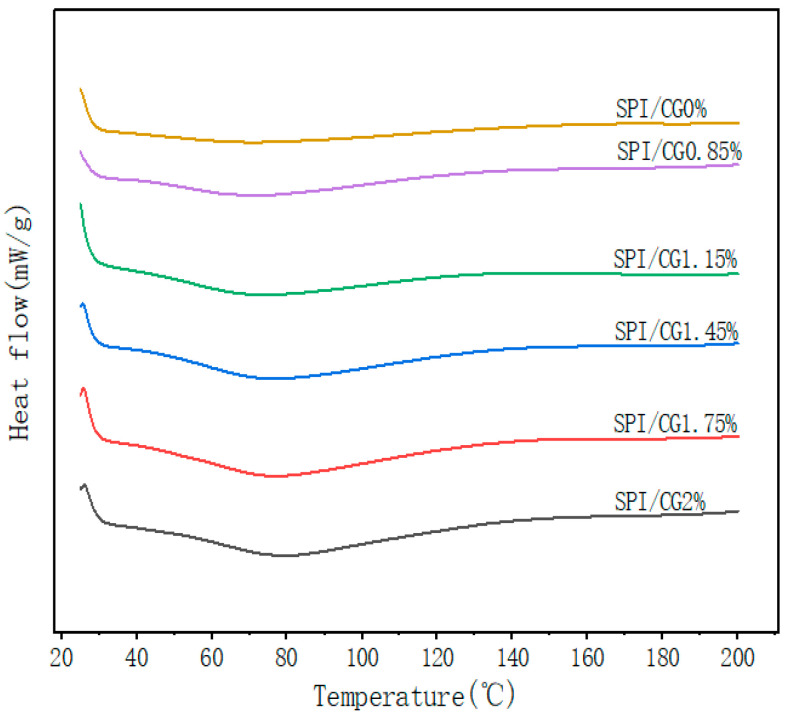
Effect of different concentrations of cassia bean gum on the thermal properties (DSC) of emulsion gels. SPI/CG0%: soy protein isolate, no cassia bean gum added; SPI/CG 0.85%: SPI + 0.85% cassia bean gum; SPI/CG 1.15%: SPI + 1.15% cassia bean gum; SPI/CG 1.45%: SPI + 1.45% cassia bean gum; SPI/CG 1.75%: SPI + 1.75% cassia bean gum; SPI/CG 2%: SPI + 2% cassia bean gum.

The influence of DSC on the thermal properties of the cassia bean gum emulsions of different concentrations was studied. The absorption peak on the thermal characteristics curve of the 0% CG/SPI emulsion gel was not obvious (Figure 5), and the denaturation temperature was 69.25 °C. As the CG concentration increased, the absorption peaks on the thermal characteristic curves of the cassia bean gum emulsion gels were evident at 69–81 °C, which was the denaturation temperature range of the emulsion gel. The absorption peak gradually moved to the right, and the denaturation temperature increased. When the CG concentration was 2%, the denaturation temperature of the emulsion gels reached 80.66 °C possibly because the interaction between cassia bean gum at a high concentration and SPI was strengthened, the oil–water interface area increased, and a stable emulsion gel structure formed. The complete denaturation of the emulsion gel required an increase in the heat absorption rate, thus improving the thermal stability of the gels [29].

### 2.7. Cooking Loss, Emulsification Stability, and pH of Meat Sausage before and after FT Treatment

The cooking loss, pH, and emulsion stability of the meat sausage before and after freezing and thawing are shown in Table 2. The pH of the meat sausage was not significantly affected by the addition of different fat replacement ratios before and after freezing and thawing (*p* ≤ 0.05), and the average pH of the emulsion gels ranged from 6.53 to 6.68. Panagiotopoulou et al. [30] demonstrated that substituting part of or all the animal fat with a Pickering emulsion did not affect the average pH value of the pork sausage; this result was consistent with the results of the present study. As the amount of emulsion gel increased, the cooking loss of meat sausage before and after freezing and thawing increased. When the amount of fat replacement was less than 50%, the cooking loss of S1 (0.04%) and S2 (0.06%) was significantly lower than that of the control group (0.07%) possibly due to the tight network structure and texture of the emulsion gel during cooking, which locked oil and water [31]. However, the rates of cooking loss in S3 and S4 were greater than the rate in the control group because water and fat were bound during heating. When the amount of fat replacement was 50%, the cooking loss was similar to that in the control group. The same trend was observed for the water seepage rate and oil permeability in all the experimental groups possibly because an appropriate amount of emulsion gel and meat protein formed the dense spatial network structure, which effectively locked water and oil. Paglarini et al. [32] found that the emulsification stability and cooking loss of emulsified sausage with a certain proportion of composite gel were close to those of the control group.

### 2.8. Color Difference Characteristics of Meat Sausage before and after Freeze–Thaw Treatment

Color is one of the most important factors for determining consumers’ preferences regarding meat products. As shown in Table 3, the color parameters brightness (L*), red (a*), and yellow (b*) of the meat sausage before and after freezing and thawing were higher than those of the control group (*p* < 0.05). The highest L* values were 63.74 and 63.81 in the S4 group. This may be because the oil droplet diameter of a meat sausage containing the emulsified gel is smaller than that produced by animal fat, which creates a greater light reflection, resulting in a higher L* [33]. The a* value of the meat sausage was not considerably different from that of the control group before and after the freeze–thaw treatment, indicating that the addition of the emulsion gel had no adverse effect on the redness value of the meat sausage. The b* value of the meat sausage before and after the freeze–thaw treatment was lower than that of the control group, showing a downward trend. The lowest values were 1.78 and 2.71 (S4), which may have been influenced by changes in the vegetable oil pigment and the emulsification of SPI after the emulsion gel was heated [34]. Li et al. [35] added laminaria polysaccharides at different concentrations into chicken sausage as a fat substitute. The L* value increased with the proportion of the substitute, and the a* value gradually decreased. Moreover, after the freeze–thaw treatment, the L* and a* values increased, and the b* values showed no considerable difference before and after the freeze–thaw treatment.

### 2.9. Texture Characteristics of Meat Sausage before and after Freeze–Thaw Treatment

TPA is an important index for evaluating the quality and overall acceptability of meat products. As shown in Table 4, as the amount of emulsion gel replacement increased, the hardness of the meat sausage before and after the freeze–thaw treatment increased and then decreased, and the hardnesses of S1 and S2 before the treatment were 9097.83 and 9745.53 N, respectively. After freezing and thawing, the hardnesses of S1 and S2 were 7643.38 and 7911.79 N. When the amount of emulsion gel was greater than 50%, the hardness decreased considerably, and the lowest hardness of S4 was obtained (5959.92 and 4850.13 N). The possible reason is that animal fat is a solid fat with a hard texture. By contrast, the emulsion gel has a soft texture. The lowest hardness of S4 was observed when the emulsion gel partially or completely replaced animal fat in meat sausage. The quality of meat products can be negatively affected [36]. Pintado et al. [37] found that when different proportions of a soybean emulsion gel were used to replace animal fat in sausage, its hardness and chewiness were be substantially reduced. The hardness of S2 before and after the freeze–thaw treatment was close to that of the control group, showing excellent freeze–thaw stability. In all the experimental groups, the chewiness, viscosity, and hardness showed the same change trend; that is, the chewiness and viscosity of S3 and S4 were significantly lower than those of C, S1, and S2 (*p* < 0.05), and the chewiness and viscosity of S2 and C were similar. This also proves that meat sausage has a lower hardness, and S2 showed excellent freeze–thaw stability. There was no significant difference in the elasticity and cohesiveness between the two groups (0.91–0.96) (*p* < 0.05).

### 2.10. Antioxidant Activity of Meat Sausage

**Figure 6:**
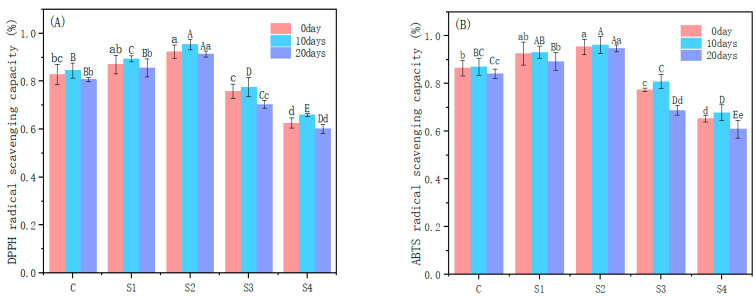
DPPH (**A**) and ABTS (**B**) free radical clearance of meat sausage during storage with different fat replacement amounts. C stands for (emulsion gel 1.75% CG/SPI) minced sausage with 0% fat replacement; S1 represents (emulsion gel 1.75% CG/SPI) meat sausage with 25% fat replacement; S2 stands for (emulsion gel 1.75% CG/SPI) minced sausage with 50% fat replacement; S3 stands for (emulsion gel 1.75% CG/SPI) minced meat with 75% fat replacement; S4 stands for (emulsion gel 1.75% CG/SPI) meat sausage with 100% fat replacement. Different letters indicate significant differences in the data (*p* < 0.05).

As shown in Figure 6, as the amount of emulsion gel added increased, the DPPH and ABTS free radical clearance rates of the meat sausage increased and then decreased. When the amount of fat replacement was 0–50%, the DPPH free radical scavenging rate increased from 83% to 92%, and the ABTS free radical scavenging rate increased from 86% to 95% possibly because the dense networks in the emulsion gel hindered the movement of the liquid oil phase and the transfer of oxidation products while inhibiting the penetration and diffusion of oxygen within the meat sausage meat sausage, thus inhibiting oil oxidation [38]. Millao et al. [39] reported that due to the strengthening of the gel structure, the antioxidant capacity of oleogel gradually increased with an increasing EC concentration. In addition, the antioxidant capacities of raw materials enhanced the antioxidant capacity of oil; that is, rapeseed oil was rich in antioxidant materials, such as vitamin E, which can remove free radicals. Therefore, the free radical scavenging rates of DPPH and ABTS were high in all the groups and increased with the amount of the emulsion gel. However, when the amount of fat replacement was greater than 50%, the free radical scavenging rates for DPPH and ABTS significantly decreased (*p* < 0.05). The scavenging rates for DPPH and ABTS decreased to 63% and 65%, respectively, because of the large amount of water lost from the meat sausage during processing after the addition of the emulsion gel. Moreover, the structure of the gel network was damaged, and the rapeseed oil was degraded by heat.

As the storage time increased, the free radical scavenging rates for DPPH and ABTS in the meat sausage increased and then decreased because of the hydrolysis of some antioxidants at the later stage of storage. The DPPH and ABTS free radical scavenging rates in S2 were the most stable during storage.

## 3. Conclusions

The effects of cassia bean gum on the texture, rheology, stability, and microstructure of the emulsion gels of a protein–polysaccharide composite matrix were investigated. As the cassia bean gum concentration increased, the texture of the emulsion gels became harder, and the surface was smooth and delicate. The size of the internal structural pores gradually decreased and was evenly distributed, and the thickness of the gel increased. Thus, the stability of the emulsion increased. During gel formation, the interaction between CG and SPI produced a large number of hydrogen and isopeptide bonds, increasing the absorption peak intensity at amides I and II in the infrared region. The presence of CG considerably improved the hardness, elasticity, and WHC of the emulsion gel. The viscosity of the emulsion gels with different concentrations of CG decreased with an increasing shear rate, showing typical pseudoplastic fluid characteristics. The G′ values of emulsion gels in frequency scanning were higher than the G″ values, and the G′ and G″ values were less dependent on the frequency. Thus, the gel network structure was stable and resilient. DSC analysis showed that the denaturation temperature of emulsion gel reached to 80.66 °C after the addition of different concentrations of CG. The 1.75% CG/SPI emulsion gel was applied to replace animal fat in meat sausage. As the amount of the replacement increased, the texture, emulsification stability, color difference, and DPPH and ABTS free radical clearance rates of the meat sausage increased first and then decreased. The freeze–thaw stability and oxidation stability of S2 were stronger than those of the control group. Therefore, the emulsion gel can replace 50% of fat for low-fat meat sausage production. This study provides a novel method for reducing the content of animal fat in meat sausage and maintaining its quality.

## 4. Materials and Methods

### 4.1. Materials

The materials used were as follows: soybean protein isolate (purity ≥ 90%): Shandong Zeenda Food Raw Materials Co., LTD. (Shandong, China); rapeseed Oil: Hunan Baling Oil Co., LTD. (Hunan, China); cassia bean gum: Shaanxi Chenming Biotechnology Co., LTD. (Shaanxi, China); potassium bromide (analytical grade): Guangdong Qianjin Chemical Reagent Co., LTD. (Guangdong, China); pork lean meat, pig backfat, and other ingredients were purchased from local shopping malls (Chengdu, China); all other reagents used in this study are analytical-grade reagents.

### 4.2. Preparation of Emulsion Gel

The emulsion gel was prepared by referring to the method of Gao et al. [40], with modifications. The soybean protein isolate powder was dispersed in deionized water (5 g/100 mL) and magnetically stirred at 6 °C at room temperature for 1.5 h to achieve a fully hydrated solution. The soybean protein isolate solution was heated to 75 °C for 15 min and then rapidly cooled in an ice bath. The concentrations of cassia bean gum used were 0%, 0.85%, 1.15%, 1.45%, 1.75%, and 2%; these were mixed with soybean protein isolate solution, and then the composite solution was mixed with rapeseed oil (19%, *w*/*w*). A high-pressure homogenizer (XHF-DY, Xinzhi Biotechnology Co., LTD., Ningbo, China) was used for homogenization at 20,000 rpm for 8 min, and the sample was then heated at 50 °C for 30 min and cooled to a gel. The final emulsion gel was left to rest overnight at 4 °C for analysis.

### 4.3. Observation of Emulsion Gel Structure

Our appearance observation occurred as follows: 30 g of the prepared fresh sample was transferred into a bottle and placed in the refrigerator at 5 °C overnight, and then it was left to stand at room temperature for 1 h before shooting, and the inverted non-flowing form was used as the standard for forming the gel.

We performed scanning electron microscopy (SEM) as follows: According to the method of Lei et al. [41], with some changes, the sample was first frozen in a freezer (−80 °C) for 8 h to fix its structure, was then put in a freeze-drying machine for 48 h for removal, and then the sample was soaked in petroleum ether for 12 h; this process was repeated 6 times, and then the degreased sample was placed in a vacuum drying oven at 70 °C for 4 h to evaporate the petroleum ether. The microstructure of the samples was observed using SEM (JSM-5800 LV, JEOL Ltd., Tokyo, Japan). The acceleration voltage was 10.0 kV, and the micromorphology of the sample was observed at 500×.

### 4.4. Determination of Physical and Chemical Properties of Emulsion Gel

#### 4.4.1. Texture Characteristics

Here, we referred to the method of Meng et al. [42] and modified it. The physical property tester (Vector 33, Bruker Optics, Ettlingen, Germany) and P36 R probe were used to determine the emulsion gel’s texture properties. Before the experiment, the emulsion gel was left to stand at room temperature at 5 °C for 2 h. The emulsion gels (3.2 cm in diameter) were tested in parallel 4 times, the strain level was 50%, and the following parameters were used for determination: the pre-test velocity, the lateral center velocity, and the post-test velocity; these were 5.0, 5.0, and 6.0 mm/s, respectively, and the contact force was 15 g. The hardness (N), elasticity, cohesiveness, and chewiness (mJ) of the emulsion gel were recorded.

#### 4.4.2. Rheological Behavior

Referring to the method of You et al. [43], with modifications, the rheological properties of the sample were determined using a rheological analyzer (MCR-101, Anton Paar Co. Ltd., Graz, Austria) with a measuring gap of 1 mm and a parallel plate diameter of 50 mm.

Temperature scanning was conducted as follows: The gel sample was placed in the rheological plate, the excess sample around the pressed plate was scraped off, and the plate was sealed with silicone oil and covered to prevent water loss at high temperature. The fixed scanning frequency was 1 Hz, the strain was 0.1%, and the change of the energy storage modulus (G′) when the gel sample was heated from 20 °C to 90 °C was recorded.

The shear rate was determined as follows: in the shear rate range of 0.01–10 s^−1^, the temperature is 25 °C, and the change of the apparent viscosity of the sample with the shear rate is recorded to determine the fluid type.

Frequency scanning was conducted as follows: The angular frequency range is 10~100 rad/s and the strain is 0.1%. Frequency scanning is performed on the emulsion gel at 25 °C, and the changes in the energy storage modulus (G′) and loss modulus (G″) with the angular frequency are recorded.

#### 4.4.3. Water Holding Capacity

According to Zhao et al.’s method [44], with slight modifications, 20 g samples were placed in a 50 mL centrifuge tube, the gels were centrifuged at 4 °C at 12,000× *g* for 15 min to remove excess water, and the surface water of the emulsion gel was absorbed using filter paper before being weighed. The water holding capacity (WHC) of the emulsion gel is calculated according to the formula:

$$WHC(\%)=\frac{W2}{W1}\times 100$$

where *W*1 is the mass of emulsion gel before centrifugation, and *W*2 is the mass of emulsion gel after centrifugation.

#### 4.4.4. FTIR Spectroscopy

Using a modified method with reference to Meng et al. [45], freeze-dried and crushed samples were mixed with potassium bromide at 1:120 and pressed into tablets for further FTIR determination (Vector 33, Bruker Optics, Ettlingen, Germany). The scanning range of infrared spectrum is 4000–400 cm^−1^, the resolution is 4 cm^−1^, and the scanning times is 32.

#### 4.4.5. Differential Scanning Calorimetry (DSC) Analysis

Referring to Zhu et al.’s [46] method, with modifications, a differential scanning calorimeter (Q200M, TA instrument, New Castle, DE, USA) was used to analyze the thermal properties of the samples. Each sample of about 5–6 mg was sealed in an aluminum crucible with a hole cover, and an empty aluminum crucible without samples was used as a reference group. The heating temperature range was 25–200 °C, and the heat distribution was measured under inert nitrogen gas at a rate of 5 °C/min and a flow rate of 10 mL/min.

### 4.5. Preparation of Meat Sausage

Here, we referred to the method of Qi et al. [47] and modified it. Each experiment consisted of five groups of meat sausages, as shown in Table 5. All laboratory meat sausages utilized the same lean meat from the front leg of the pig, along with consistent backfat and toppings, to minimize variations between batches. The pork front leg meat underwent a pre-treatment process in which the visible fasciae were removed and the meat was minced. Subsequently, the minced meat was combined with an appropriate amount of salt, sodium ascorbate, and sodium tripolyphosphate to facilitate the dissolution of the meat proteins. After marinating for eight hours, the mixture was blended at a high speed for approximately 20 min until a strong texture was achieved, during which 30 g of cold water was added in batches. Following this, the cured minced meat, pork fat, or emulsion gel (with fat replacement ratios of 0%, 25%, 50%, 75%, and 100%) was stirred in a food processor for five hours. The resulting meat sausage mixture was then steamed in a pot at 60 °C for 30 min, rapidly cooled in an ice bath, and stored at 4 °C for further analysis.

### 4.6. Quality Determination of Meat Sausage before and after FT Treatment

The samples were frozen in a constant temperature freezer at −18 °C for 24 h. After freezing, the samples were thawed in a constant temperature refrigerator at 4 °C for testing.

#### 4.6.1. Determination of Cooking Loss

About 15 g of minced meat samples were weighed and heated at 90 °C for 30 min. After cooling in a cold bath for 30 min, the surface moisture of the sample was absorbed with filter paper to record the quality of the minced meat before and after cooking. The cooking loss of minced meat was calculated according to the formula:

$$\mathrm{Cooking}\;\mathrm{loss}\;(\%)=\frac{M1-M2}{M1}\times 100$$

where *M*1 is the weight of the minced meat before cooking, and *M*2 is the weight of the minced meat after cooking.

#### 4.6.2. Determination of Emulsification Stability

In accordance with the modifications suggested by Pan et al. [48], 20 g of raw minced meat was placed into a 50 mL centrifuge tube, which was then heated in a water bath at 80 °C for 20 min. After heating, the tube was removed and allowed to cool before being centrifuged at a centrifugal force of 2500× *g* for 3 min. The centrifuge tube was subsequently inverted and placed in a beaker for 1 h. Following this, the solid portion of the sample was extracted, weighed, and dried in an oven at 100 °C for 10 h to achieve a constant weight. The water permeability (*W*) and fat permeability (*F*) of the minced meat were calculated using the appropriate formulas:

$$W(\%)=\frac{M2-M3}{M1}\times 100$$

$$F(\%)=\frac{M3-M4}{M1}\times 100$$

where *M*1 is the quality of the raw meat, *M*2 is the quality before drying, *M*3 is the quality after drying, and *M*4 is the quality of the empty bottle.

#### 4.6.3. Measurement of pH Value

We referred to the method of Yu et al. [49] to determine the pH value of meat sausage. We took 5 g of the cut sausage sample, added 50 mL distilled water to homogenize it for 5 min, then let it stand for 30 min, and removed the supernatant to determine the pH value.

#### 4.6.4. Determination of Color Difference

According to the method of Cheng et al. [50], with modifications, the color of the meat sausage was determined. The meat sausage was cut into a flat and smooth cylinder with a section height of 2 cm, corrected with a standard blackboard and whiteboard, and the observer angle was measured to be 0°. Using the three color values (brightness, L* value; redness, a* value; yellowness, b* value), each sample was measured 6 times in parallel.

#### 4.6.5. Determination of Texture

The method of Wang et al. [51], with slight modifications, was used to determine the texture of the meat sausage. Meat sausage samples were cut into cylinders with a diameter of 20 mm and a height of 20 mm, and the texture analysis of meat sausage samples was performed with a physical property tester (Vector 33, Bruker Optics, Ettlingen, Germany). A p/50 probe was equipped to perform a two-cycle axial compression test with a stress of 30%. The test speeds before, during, and after the test were 5.0 mm/s, 5.0 mm/s, 1.0 mm/s, and the trigger force was 5.0 g, respectively. The parameters measured were hardness (g), elasticity, cohesion, chewiness (mJ), and adhesion.

### 4.7. Determination of Antioxidant Activity of Meat Sausage

According to the method of Feng et al. [52], although slightly modified, 10 g of cut meat sausage was placed into 50 mL of distilled water and homogenized in a water bath at 30 °C for 1 h, and 0.2 mL of filtrate was mixed with 1 mL of DPPH solution (0.1 mM, diluted with anhydrous ethanol). After 30 min of light-avoidance reaction at room temperature, the absorbance at 517 nm was measured as the experimental group. Also, 0.2 mL of distilled water and 1 mL of DPPH solution were used as the control group, and 0.2 mL of filtrate and 1 mL of anhydrous ethanol were used as the blank group. The DPPH radical scavenging capacity of the meat sausage was calculated according to formula:

$$\mathrm{DPPH}\;\mathrm{radical}\;\mathrm{scavenging}\;\mathrm{capacity}\;(\%)=\frac{B1-(B2-B3)}{B1}\times 100$$

where *B*1 is the absorbance at 517 nm of the control group, *B*2 is the absorbance at 517 nm of the experimental group, and *B*3 is the absorbance at 517 nm of the blank group.

According to the method of Kong et al. [53], although slightly modified, the mother liquor of ABTS was obtained by mixing 200 mg of ABTS with 34 mg of potassium persulfate and 50 mL of distilled water, shaking well, and leaving overnight at room temperature away from light. The ABTS solution, with an absorbance 0.7 ± 0.02 at 734 nm, was prepared by diluting the ABTS mother solution to a certain multiple with 95% anhydrous ethanol. The absorbance at 734 nm was measured by mixing 25 μL of filtrate and 1 mL of ABTS solution at room temperature for 10 min after the reaction. Here, 25 μL of distilled water and 1 mL of ABTS solution were used as the blank group. The ABTS radical scavenging capacity of the meat sausage was calculated according to formula:

$$\mathrm{ABTS}\;\mathrm{radical}\;\mathrm{scavenging}\;\mathrm{capacity}\;(\%)=\frac{\mathrm{Bblank}-\mathrm{Bsample}}{\mathrm{Bsample}}\times 100$$

where *Bsample* is the absorbance of the experimental group at 734 nm, and *Bblank* is the absorbance of the blank group at 734 nm.

### 4.8. Data Analysis

All tests were carried out in triplicate, and the data were presented as the mean ± SD (standard deviation). SPSS software (26.0 for Mac, IBM SPSS Statistical software Inc., Chicago, IL, USA) was used for statistical analysis. ANOVA and Duncan’s multiple range tests were used for statistical analysis. The significance level for all tests was established at *p* ≤ 0.05.

## Figures and Tables

**Table 1:** Texture characteristics of emulsion gels.

Groups	Hardness (N)	Elasticity	Cohesiveness	Chewiness (mJ)
SPI/CG 0%	43.67 ± 0.16 f	0.99 ± 0.02 a	0.98 ± 0.01 a	43.53 ± 0.16 f
SPI/CG 0.85%	131.68 ± 1.22 e	0.98 ± 0.01 ab	0.91 ± 0.01 b	115.35 ± 4.16 e
SPI/CG 1.15%	221.50 ± 3.10 d	0.95 ± 0.01 c	0.82 ± 0.01 c	167.26 ± 8.16 d
SPI/CG 1.45%	384.67 ± 6.34 c	0.94 ± 0.01 c	0.81 ± 0.01 c	294.79 ± 4.41 c
SPI/CG 1.75%	586.91 ± 11.84 b	0.94 ± 0.01 bc	0.81 ± 0.01 c	452.94 ± 9.77 a
SPI/CG 2%	558.94 ± 9.14 a	0.93 ± 0.01 bc	0.80 ± 0.01 d	399.89 ± 7.23 b

Data are expressed as the mean ± standard deviation. a–f: different lowercase letters indicate significant differences (*p* < 0.05). SPI/CG0%: soy protein isolate, no cassia bean gum added; SPI/CG 0.85%: SPI + 0.85% cassia bean gum; SPI/CG 1.15%: SPI + 1.15% cassia bean gum; SPI/CG 1.45%: SPI + 1.45% cassia bean gum; SPI/CG 1.75%: SPI + 1.75% cassia bean gum; SPI/CG 2%: SPI + 2% cassia bean gum.

**Table 2:** Cooking loss, emulsification stability, and pH of meat sausage before and after FT treatment.

Groups	Cooking Loss	Before FT Treatment			After FT Treatment		
		PH	W	F	PH	W	F
C	0.07 ± 0.002 c	6.56 ± 0.02 c	0.046 ± 0.004 c	0.01 ± 0.008 c	6.53 ± 0.04 d	0.058 ± 0.001 c	0.028 ± 0.004 c
S1	0.04 ± 0.002 e	6.61 ± 0.02 b	0.041 ± 0.007 c	0.011 ± 0.003 bc	6.55 ± 0.01 c	0.046 ± 0.003 d	0.026 ± 0.003 d
S2	0.06 ± 0.002 d	6.62 ± 0.04 d	0.047 ± 0.005 c	0.017 ± 0.006 ab	6.57 ± 0.02 c	0.056 ± 0.004 c	0.031 ± 0.005 c
S3	0.09 ± 0.003 b	6.63 ± 0.01 b	0.073 ± 0.006 b	0.02 ± 0.005 ab	6.59 ± 0.01 b	0.074 ± 0.008 b	0.04 ± 0.005 b
S4	0.1 ± 0.001 a	6.68 ± 0.02 a	0.085 ± 0.001 a	0.022 ± 0.002 a	6.63 ± 0.02 a	0.096 ± 0.002 a	0.053 ± 0.002 a

Data are expressed as the mean ± standard deviation. FT, freeze–thaw; cook loss; cook loss; W, water permeability; F, oil permeability. a–e: different lowercase letters indicate significant differences (*p* < 0.05). C stands for (emulsion gel 1.75% CG/SPI) minced sausage with 0% fat replacement; S1 represents (emulsion gel 1.75% CG/SPI) meat sausage with 25% fat replacement; S2 stands for (emulsion gel 1.75% CG/SPI) minced sausage with 50% fat replacement; S3 stands for (emulsion gel 1.75% CG/SPI) minced meat with 75% fat replacement; S4 stands for (emulsion gel 1.75% CG/SPI) meat sausage with 100% fat replacement.

**Table 3:** Color difference in minced meat before and after FT treatment.

Groups	Before FT Treatment			After FT Treatment		
	L*	a*	b*	L*	a*	b*
C	60.55 ± 0.17 e	15.78 ± 0.13 a	3.11 ± 0.03 a	61.59 ± 0.16 e	15.86 ± 0.11 a	3.77 ± 0.12 a
S1	61.45 ± 0.16 d	15.56 ± 0.08 b	2.84 ± 0.13 b	62.04 ± 0.04 d	15.64 ± 0.05 c	3.63 ± 0.04 ab
S2	61.78 ± 0.05 c	15.49 ± 0.02 bc	2.34 ± 0.09 c	62.81 ± 0.14 c	15.61 ± 0.26 ab	3.49 ± 0.11 b
S3	62.63 ± 0.13 b	15.37 ± 0.08 c	1.90 ± 0.09 d	63.51 ± 0.10 b	15.42 ± 0.17 b	2.92 ± 0.04 c
S4	63.74 ± 0.12 a	15.31 ± 0.14 c	1.78 ± 0.06 d	63.81 ± 0.06 a	15.40 ± 0.19 b	2.71 ± 0.15 d

Data are expressed as the mean ± standard deviation. FT, freeze–thaw; L*, brightness; a*, red; b*, yellow. a–e: different lowercase letters indicate significant differences (*p* < 0.05). C stands for (emulsion gel 1.75% CG/SPI) minced sausage with 0% fat replacement; S1 represents (emulsion gel 1.75% CG/SPI) meat sausage with 25% fat replacement; S2 stands for (emulsion gel 1.75% CG/SPI) minced sausage with 50% fat replacement; S3 stands for (emulsion gel 1.75% CG/SPI) minced meat with 75% fat replacement; S4 stands for (emulsion gel 1.75% CG/SPI) meat sausage with 100% fat replacement.

**Table 4:** Texture characteristics of meat sausage before and after FT treatment.

Groups	Before FT Treatment					After FT Treatment				
	Hardness (N)	Elasticity	Cohesiveness	Adhesiveness (N-mm)	Chewiness (mJ)	Hardness (N)	Elasticity	Cohesiveness	Adhesiveness (N-mm)	Chewiness (mJ)
C	9897.42 ± 107.7 a	0.95 ± 0.01	0.94 ± 0.02	9413.59 ± 76.09 a	8831.74 ± 107.64 a	7643.38 ± 57.28 b	0.94 ± 0.02	0.94 ± 0.02	7248.75 ± 131.14 a	6905.06 ± 73.94 a
S1	9097.83 ± 65.34 b	0.96 ± 0.01	0.95 ± 0.01	8612.65 ± 44.14 b	8268.27 ± 121.65 c	7911.79 ± 111.77 a	0.95 ± 0.01	0.91 ± 0.06	6532.66 ± 92.26 b	6108.69 ± 72.63 b
S2	9745.53 ± 55.31 c	0.96 ± 0.01	0.95 ± 0.01	9131.36 ± 83.81 c	8989.2 ± 83.95 b	7860.28 ± 99.01 a	0.94 ± 0.01	0.91 ± 0.05	7169.74 ± 292.5 a	6878.2 ± 84.31 a
S3	7236.98 ± 22.04 d	0.95 ± 0.02	0.95 ± 0.01	6907.28 ± 64.29 d	6566.53 ± 53.83 d	5236.98 ± 22.04 c	0.93 ± 0.01	0.92 ± 0.01	5207.28 ± 64.29 c	5033.2 ± 33.25 d
S4	5969.92 ± 43.49 e	0.95 ± 0.01	0.92 ± 0.01	5471.42 ± 113.59 e	5195.68 ± 101.17 e	4850.13 ± 60.66 d	0.93 ± 0.01	0.92 ± 0.01	4552.31 ± 55.05 d	4394.4 ± 65.19 c

Data are expressed as the mean ± standard deviation. FT, freeze–thaw. a–e: different lowercase letters indicate significant differences (*p* < 0.05). C stands for (emulsion gel 1.75% CG/SPI) minced sausage with 0% fat replacement; S1 represents (emulsion gel 1.75% CG/SPI) meat sausage with 25% fat replacement; S2 stands for (emulsion gel 1.75% CG/SPI) minced sausage with 50% fat replacement; S3 stands for (emulsion gel 1.75% CG/SPI) minced meat with 75% fat replacement; S4 stands for (emulsion gel 1.75% CG/SPI) meat sausage with 100% fat replacement.

**Table 5:** List of ingredients for meat sausage preparation.

Component	Groups (Unit: g/100 g)				
	C	S1	S2	S3	S4
Forehock	70	70	70	70	70
Pork backfat	30	22.5	15	7.5	0
Emulsion gel	0	7.5	15	22.5	30
Ice water	25	25	25	25	25
Salt	3	3	3	3	3
Sugar	1	1	1	1	1
Sodium tripolyphosphate	0.2	0.2	0.2	0.2	0.2
natrascorb	0.1	0.1	0.1	0.1	0.1
Other ingredients	0.7	0.7	0.7	0.7	0.7

C stands for (emulsion gel 2% CG/SPI) minced sausage with 0% fat replacement; S1 represents (emulsion gel 2% CG/SPI) meat sausage with 25% fat replacement; S2 represents (emulsion gel 2% CG/SPI) minced meat with 50% fat replacement; S3 stands for (emulsion gel 2% CG/SPI) minced meat with 75% fat replacement; S4 stands for (emulsion gel 2% CG/SPI) meat sausage with 100% fat replacement.

## Data Availability

The data presented in this study are available on request from the corresponding author.
